# The effect of vitamin D on COPD exacerbation: a double blind randomized placebo-controlled parallel clinical trial

**DOI:** 10.1186/s40200-016-0257-3

**Published:** 2016-08-26

**Authors:** Mojgan Sanjari, Akbar Soltani, Abdolrahim Habibi Khorasani, Maryam Zareinejad

**Affiliations:** 1 Endocrinology and Metabolism Research Center, Basic and Clinical Physiology Institute, Kerman University of Medical Sciences, Kerman, Iran; 2 Endocrinology and Metabolism Research Center, Endocrinology and Metabolism Clinical Sciences Institute, EBM Group, Tehran University of Medical Sciences, Tehran, Iran; 3 Department of Pulmonary Medicine, Kerman University of medical sciences, Kerman, Iran; 4 Physiology Research Center, Institute of Neuropharmacology, Kerman University of Medical Sciences, Kerman, Iran

**Keywords:** Calcitriol, COPD, Forced expiratory volume, Forced Vital Capacity, Pulmonary function tests, Vitamin D

## Abstract

**Background:**

To investigate the effect of supplementation of standard treatment (inhaled long-acting β2 agonists, anticholinergics and corticosteroids) with vitamin D on C reactive protein and pulmonary function tests in patients with COPD exacerbation.

**Methods:**

*Design:* Randomized, single-center, double-blind, placebo-controlled parallel trial. *One teaching hospital Participants:* 135 patients in pulmonary ward with moderate to severe COPD and exacerbations.120 patients fulfilled the study protocol. *Interventions:* Patients were randomly divided into three groups receiving 7 day treatment with 0.25 μg calcitriol daily (*n* = 45), 50000 IU daily of vitamin D (*n* = 45) or placebo (*n* = 45). An independent nurse was responsible for allocation, preparation, and accounting of trial medications. *Main Outcome measures:* Maximal expiratory flow volume (FEV1) and forced volume capacity curves (FVC) and Modified Medical Research Council (MMRC) scale.

**Results:**

Out of 135 patients who were recruited consecutively, 45 patients randomly were randomly assigned in three groups (balance blocked randomization.15 patients were dropped out due to non-compliance for second PFT. Intention to treat analysis was carried out for 120 participants. The difference between before and after treatment FEV1 and FEV1/FVC ratio had no significant difference between treatment groups and placebo. (*P* = 0.43, *P* = 0.51, respectively)but clinical improvement was significant in patients who received calcitriol. No side effects were reported.

**Conclusions:**

Short term treatment with either calcitriol or 25(OH) _2_Vit D didn’t changed FEV1 or FVC in vitamin D sufficient patients with COPD exacerbation; nevertheless it can provide clinical benefit.

**Trial registration:**

Trial registration: Iranian Registry of Clinical Trials no. IRCT138712271774N1. Registered 10 April 2011.

## Background

Chronic obstructive pulmonary disease (COPD) is the fourth main cause of death in the United States [1]. Many studies have shown 25-OHD serum levels in patients suffering from COPD.

Vitamin D deficiency in African-Americans is closely associated with higher susceptibility to COPD [2, 3]. Also, the studies suggest that there is a link between genetic variants in the vitamin D pathway and COPD. For example, studies suggest that a single nucleotide polymorphism (SNP) of the vitamin D binding protein protects COPD through a mechanism that is still unknown to researches [4].

The VDR levels is significantly low in lung tissues of COPD patients [5]. Active metabolite of vitamin D (1, 25 dihydroxy vitamin D3) has a vital role in cellular metabolism and differentiation by means of its nuclear receptor (VDR) that is associated with a number of other chromatin modification enzymes (histone acetyltrans-ferases and histone deacetylases), and therefore involved in the complex epigenetic events in vitamin D signaling and metabolism [6].

Recently, studies have found a linear correlation between higher risks of vitamin D deficiency in patients with COPD and the function of lung and systemic levels of vitamin [7, 8]. Nevertheless in a cohort study carried out on patients with severe COPD, baseline 25 (OH) D levels could not predict the ensuing acute exacerbation of COPD [8]. Patients with COPD are subjected to exacerbation. The symptoms of exacerbations are normally severe deterioration of symptoms, intense airway inflammation and physiological worsening [9]. They are assumed to act as a reference in the therapeutic study of COPD. Recently, some studies have found the effect of exacerbations on disease progression, which can be manifested by accelerated forced expiratory volume in one second (FEV1), and reduced characteristic of COPD [10, 11]. Brehm showed that vitamin D deficiency is related to severe of allergy and asthma attacks. There was a significant and inverse relationship between the level of Serum vitamin D and increased airway responsiveness [12]. Exacerbations is estimated to be the cause of nearly 25 % of the FEV1 decline in COPD [10].

With the exception of smoking quitting, no other proved treatment has been found for hindering COPD disease progression; however, two recent large randomized controlled experiments have shown the positive impacts of long-acting bronchodilators on the rate of exacerbation, life quality, and survival [11, 13].

It is while the inefficacy of a host of new therapeutics or recombinant antibodies have been proved. In light of the unsatisfactory results of these expensive medications, greater attention should be paid to the potential of traditional medications. The potential therapeutic effect of drugs such as statins, angiotensin-converting enzyme (ACE)–inhibitors, and heparin have not been recognized and they may attract greater attention in future therapy of COPD [14, 15]. This study explores the potentials of another, even older molecule, vitamin D.

A cross-sectional, retrospective study of children there is a relationship between in Costa Rica show that vitamin D deficiency and higher symptoms of asthma severity [12]. Another study suggested the higher risk of severe asthma exacerbation in patients suffering from vitamin D insufficiency [16]. The aim of this study is to evaluate the role of exogenous addition of vitamin D and 1, 25 dihydroxy vitamin D on the function of lung in the patients suffering from COPD exacerbation.

## Methods

Study Design and Participants: The current study was a single-center, double-blind research, which used parallel group and randomized placebo-controlled trial. Two hundred and five patient were assessed for eligibility (Not meeting inclusion criteria, *n* = 38 & Declined to participate, *n* = 32). A total of 135 patients aged between 40 and 60 years were recruited consecutively diagnosed as having COPD from the emergency department of Kerman Afzalipour university Hospital in Kerman city (the largest province in south east of Iran) during winter of 2011. All subjects were ex-smokers with a history of chronic cough and expectoration who also had exertional dyspnea and were admitted with the diagnosis of COPD exacerbation.

Based on the ERS criteria, COPD defined when FEV1 % prediction is <88 % for men, or <89 % for women [17, 18]. An acute exacerbation of COPD can be defined as a dramatic degradation of COPD symptoms (for example, the quantity and the color of phlegm or shortness of breath) that last for a couple of days [19].

Patients were excluded if the following were presented: unwillingness to participate in the study, unable to perform spirometry, A history of asthma symptoms, existence of other respiratory disorders including bronchial carcinoma, a history of hospitalization (within 4 weeks) for COPD, any medical condition which needed more invasive respiratory support, symptoms of lower respiratory tract infection or other kinds of simultaneous systemic disease such as hypercalcemia, renal failure, hyperparathyroidism, malignancy, history of renal stone, cardiac arrhythmia, or patients who were using lithium.

**Fig. 1:**
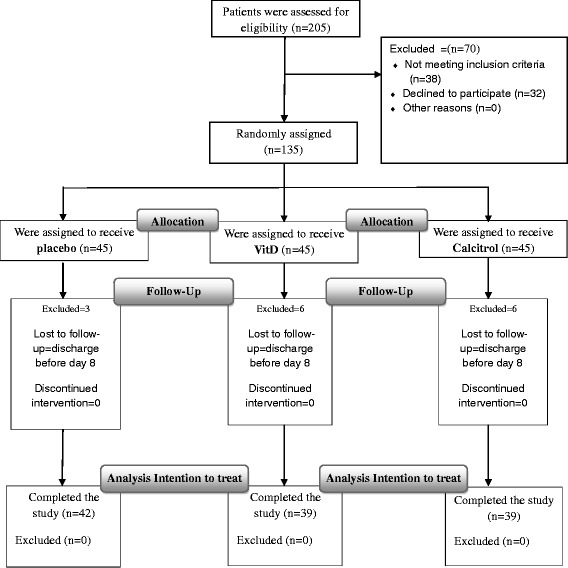
Study flow diagram

All subjects signed an informed written consent to participate in the study. The protocols of the study were confirmed by the ethics committee of KUMS [Kerman university of medical sciences (Code 88/17)], and this trial was registered on the IRCT [Iranian Registry of Clinical Trials] website (the registration number of the trial is IRCT138712271774N1). We tried this randomized controlled trials conform to the CONSORT statement and flow diagram (Fig. 1) [19].

Full trial protocol can be accessed in Iranian Registry of Clinical Trials website.

Randomization and Masking: In this study, we used a double-blind randomized, parallel group, placebo-controlled design. After the preliminary evaluation, the qualified patients were randomly assigned to one of the three treatment groups and followed up for one week. Participants were randomized by a computer-generated list to 3 equal groups. Patients were divided into 3 groups according to the balance blocked randomization. Out of 135 patients, 45 patients randomly were selected in three groups of A, B and C. Patients in each group received same envelopes containing different drugs. Both patients and the physicians and epidemiologist were blinded to the treatment received. A nurse who was not involved in care of the trial patients and independent of the site investigator was responsible for allocation, preparation, and accounting of trial medications. The trial medications and placebo was prepared at a separate site, and then taken to the clinic every week. The randomization schedule was thus concealed from all care providers, ward physicians, and other research personnel. The patients who left the study or for whom the experiment could not be completed were excluded, and finally3, 6 and 6 patients were eliminated in groups A, B and C respectively. No side effect or harm was reported.

In phone recall all of eliminated patients were alive and improved clinically after one week but they didn’t participate to the last part of the study (Second PFT and Lab Tests) due to personal problems.

### Procedures

After randomizing the patients, there were follow-up visits at the beginning and the end of study (day 0 and 8). Patient received the following treatment. Group A: Placebo (*n* = 42) - The patients in this group received standard treatment for COPD according to the guidelines of GOLD [20].

Also, these patients received placebo capsules, which were similar to active drug in group B and C, daily for a week.

Group B: Treatment with vitamin D supplementation (*N =* 39. Similar to in group A, these patients received standard treatment for COPD in addition to25 (OH)-D3 (Pourateb, Iran) 50000 IU capsules Daily for seven days. Group C: Treatment supplemented with calcitriol (*n* = 39).

Standard COPD treatment was given to these patients according to the guidelines of GOLD [20, 21] plus calcitriol (Pourateb, Iran) 0.25 μg capsules daily, for seven days.

If necessary, inhaled salbutamol was permitted. Also, to control exacerbations, additional bronchodilators, oral steroids and antibiotics were used.

Corticosteroid therapy (oral or inhaled), if present, could continue without any change. Spirometry: Spirometry was carried out at the beginning and the end of study (day 0 and 8).

The experiment was carried out on a dry, rolling-seal spirometer Benchmark model (Spirolab, UK).

According to the guidelines of American Thoracic Society/European Respiratory Society Maximal expiratory flow volume and forced volume capacity curves were attained. For each subject, three acceptable and a minimum of two reproducible curves were obtained. The greatest values of FEV1 and FVC were chosen for analysis. we used the Modified Medical Research Council (MMRC) scale for the evaluation of dyspnea in everyday activities at day 0 and 8 in each group [22] [Table 1].

**Table 1:** The modified Medical Research Council dyspnea scale (MMRC)

Grade of dyspnea Symptom	
Grade 0	Not troubled by breathlessness except on strenuous exercise
Grade 1	Short of breath when hurrying or walking up a slight hill
Grade 2	Walks slower than contemporaries on the level because of breathlessness or has to stop for breath when walking at own pace
Grade 3	Stops for breath after walking 100 m or after a few minutes on the level
Grade 4	Too breathless to leave the house or breathless when dressing or undressing

Biochemical estimations: All serum samples were stored frozen at −80 C. Serum calcium was measured for excluding hypercalcemic patients and albumin was checked for interpreting the calcium results. C-reactive protein (CRP) was measured when the follow-up period was terminated. High sensitivity CRP concentrations were measured on a Hitachi 911 analyzer by a latex-enhanced immuneturbidimetric assay from Roche. Circulating25 (OH) D3, and1, 25(OH) 2VITD was measured by Stat Fax Instrument and Immunodiagnostic System Kit using an Enzyme immunoassay method.

In each set of samples, we measured two internal control specimens that the reagent manufacturer had provided. The total variation coefficient of for measured internal controls was 9.3.

In the final stage of treatment, responses were assessed by a physician, who asked patients about the improvement, stability or deterioration of their condition.

The primary end points were FEV1 and FVC and clinical response. Secondary end points were serum level of C reactive protein, totalanti-oxidant, 25 and 1&25OH- vitamin D.

Statistical Analysis: For convenience, STATA statistical software was used for determining the sample size.
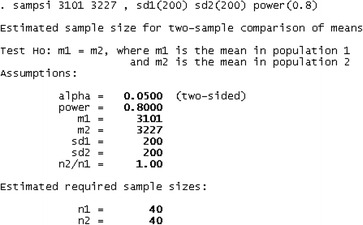


SPSS 16 (Chicago, IL) was used for statistical analysis. Paired t test and Mann Whitney test were respectively used for data with and without normal distribution. To compare categorical variables within three groups, Chi square test (and Fisher exact test if required) was used. Similarly the changes in the outcome parameters were compared by univariate analysis of variance (ANOVA) and followed by Bonferroni correction. To control the effects of first value FEV1 and FEV1/FVC, two-way ANOVA (ANCOVA) was used. All collected data were included in the intention to treat analysis.

The statistical significance level was considered less than 0.05 (*P* value < 0.05).

## Results

One hundred and twenty patients who had COPD exacerbation met the eligibility criteria and completed the trial. The details of the population are summarized in [Table 2]. Statistically, treatment arms were not significantly different with respect to age and gender and severity of disease which is shown pre-treatment FEV1 and FEV1/FVC ratio. There was not a significant statistical difference between Calcium and Albumin and C reactive protein (*P* value > 0.05). At the end of study, none of the patient developed hypercalcemia [Table 3].

**Table 2:** Baseline characteristics of the study population

Variables	Placebo (*n* = 42)	Vitamin D (*n* = 39)	Calcitriol (*n* = 39)	*P* value
Age,				
	58.4 ± 9.5	55.8 ± 9.5	55.6 ± 10.4	0.382
Sex, %				
Male	69.2	71.4	87.2	0.128
Female	30.8	28.6	12.8	
FEV_1_				
Baseline	46.7 ± 20.4	45.3 ± 18.9	46.3 ± 17.5	0.439
End	62.3 ± 22.9	64.5 ± 22.8	64.1 ± 21.3	
FEV1/FVC				
Baseline	70.2 ± 16.9	68.2 ± 16.9	69.1 ± 17.2	0.513
End	72.9 ± 14.1	73.1 ± 14.2	74.4 ± 15.4	
MMRC				
	3.62 ± 0.49	3.69 ± 0.46	3.6 ± 0.49*	0.001
	2.66 ± 0.74	2.65 ± 0.67	1.98 ± 0.65	

Data are expressed as mean ± SD

*different from placebo *p* ≤ 0.05

**Table 3:** Effect of treatments on Calcium, Albumin,CRP and different forms of vitamin D

Variables	Placebo (*n* = 42)	Vitamin D (*n* = 39)	Calcitriol (*n* = 39)	*P*. Value
Albumin (g/l)				
Baseline	39.05 ± 4.37	38.71 ± 5.7	39.7 ± 4.9	0.66
Post-Baseline(End)	43.2 ± 4.1	42.1 ± 4.2	43.4 ± 5.1	0.33
Difference (change)	4.2 ± 3.9	3.4 ± 3.7	3.76 ± 4.8	0.67
Calcium (mmol/l)				
Baseline	2.4 ± 0.19	2.46 ± 0.13	2.43 ± 0.18	0.43
Post-Baseline(End)	2.6 ± 0.15	2.6 ± 0.12	2.6 ± 0.12	0.076
Difference (change)	0.19 ± 0.18	0.20 ± 0.16	0.17 ± 0.14	0.78
CRP^a^ (Mg/dL)				
Baseline	70.1 ± 61.5	70.9 ± 52.1	67.3 ± 57.1	0.95
25OH-D(nmol/l)				
Baseline	59.9 ± 26	58.9 ± 27	54.9 ± 34.9	<0.001
End	65.2 ± 39.2	97.7 ± 52.2*	57.1 ± 44.4	
1,25OH-D(pmol/l)				
Baseline	91. ±13.5	110.2 ± 71.2	103.5 ± 83.7	0.563
End	108.7 ± 59.5	128.2 ± 121.9	101.9 ± 81.1	

^a^CRP:Creactive protein

*P < 0.001

The baseline-anticipatedFEV1 percent was the same in the three groups (Table 2). The FEV1 percent predicted in Calcitroil group showed an increase from 46.3 ± 17.5 at baseline to 64.1 ± 21.3 after treatment (*P* < 0.01). In Vitamin D group, it rose from 45. 3 ± 18.9 to 64.5 ± 22.8 after treatment (*P* < 0.01), and in Placebo group, 46.7 ± 20.4 reached to 62.3 ± 22.9 (*P* < 0.01). The difference between before and after treatment FEV1 and FEV1/FVC ratio was calculated and compared between groups by ANOVA and it no significant difference was observed between treatment groups and placebo.(*P* = 0.43, *P* = 0.51, respectively) After adjusting the effect of baseline FEV1 and FEV1/FVC ratio by ANCOVA the result didn’t changed too (*P* = 0.44, *P* = 0.54, respectively). The magnitude of increase in FEV1 and FEV1/FVC ratio was corresponding in the three groups. However, clinically improved lung function(Reduction in MMRC) was observed in all cases and was significant in the majority of patients. The MMRC in Calcitroil group showed a significant decrease from 3.6 ± 0.49 at baseline to 1.98 ± 0.65after treatment (*P* < 0.0001). In Vitamin D group, it decreased from 3.69 ± 0,46 to 2.65 ± 0.67 after treatment (*P* < 0.001), and in Placebo group, 3.62 ± 0.49reached to 2.62 ± 0.74 (*P* < 0.01). The difference between before and after treatment MMRC was calculated and compared between groups by ANOVA and Tukey test and significant difference was observed between calcitriol group vs. placebo (1.46 vs. 0.71 *P* < 0.001). Maximum reduction in MMRC was seen in calcitriol group and then vitamin D and placebo group respectively (*P* < 0.001).

Statistically speaking, there was no relationship between FEV1 and changed FEV1with serum level of 25 OH-vitamin D and 1,25 (OH)2 vitamin D before and after treatment (*P* > 0.1) there was a significant rise in the levels of plasma vitamin 25OH-D in Vitamin D group from58.9 ± 27 nmol/l at baseline to 97.7 ± 52.2 nmol/l after treatment (*P* < 0.001). In Calcitriol group, however, the variation from 54.9 ± 34.9 to 57.1 ± 44.4 nmol/l was not significant. The same result was seen in placebo group. The plasma level of 1,25OH-D was not affected significantly by any of treatments [Table 3].

## Discussion

This study revealed that short term treatment with neither calcitriol nor vitamin D has any effect on pulmonary function of COPD patients during exacerbation, nevertheless the MMRC in Calcitroil group showed a significant decrease. The level of calcitriol did not change during this study, but the increase in 25(OH) vitamin D in group B indicated the compliance of the patient in ingesting the drug.

A number of studies have stated 25-OHD serum levels in COPD patients. One recently published study showed that aspiratory muscle strength and maximal oxygen uptake in patients receiving vitamin D improved significantly compared with placebo group, but two groups were not statistically different in terms of improved quadriceps strength or six minutes walking distance [23].

Also, high doses of vitamin D(100 000 IU every 4 weeks for 1 year) in vitamin D sufficient patients did not have a significant effect on mean time before the appearance of the first exacerbation, exacerbation rates, FEV1, hospitalization, life quality, and death. In participants who had acute vitamin D deficiency at baseline, the use of supplementation could reduce exacerbations [21].

The studies of Forli and colleagues showed that more than 50 % of patients enlisted for lung transplantation had vitamin D deficiency (<20 ng/ml) [24]. Likewise, a study on community-dwelling patients in Denmark who suffered from COPD revealed 68 % of the participants had untreated osteoporosis or osteopenia [25]. It is worth noting that insufficient or low levels of vitamin D are not particular to COPD. Patients with COPD are more likely to have vitamin D deficiency for several reasons [3]. Vitamin D deficiency is a common problem in most population yet [26, 27].

The current supplementation with a daily dose of 800 to 1,000 IU of vitamin D makes up for low levels of serum 25-OHD in the majority of adults in concentrations greater than 20 ng/ml, but higher doses may be needed to increase 25-OHD levels proportional to the needs of high-risk patients who don’t have hypocalcaemia [28]. One recent study suggest that “the current vitamin D recommended dietary allowance (RDA) (600 IU/day) is insufficient to cover the skeletal health needs of at least 50 % of black and white children” [29].

In this study most patients were vitamin D sufficient maybe due to extra use of vitamin D supplementation in old population in our country [30]. It was shown that “ Vitamin D intake decreased COPD exacerbation and improved FEV1 in the patients with severe and very severe COPD” [31]. Unfortunately in this study which was carried out in our country the baseline level of vitamin D was not determined. It was shown that “vitamin D3 was protective against moderate or severe exacerbation in participants with baseline serum 25-hydroxyvitamin D concentrations of less than 50 nmol/L” too [32].

Our study showed no significant relationship between vitamin D level and FEV1 and or FEV1/FVC, it may be due to sufficient leveled vitamin D in the population of our study. Unfortunately there were not enough vitamin D deficient patients to do any statistical analysis in this subgroup. The present study showed that any relationship between vitamin D level and FEV1 or airway responsiveness may be due to high levels of vitamin D in the patients. The more important cause can be explained by the difference between the pathophysiology of asthma and chronic obstructive lung diseases a non-reversible airway disease. The level of CRP didn`t significant difference between three groups it may emphasis that this dose and duration of vitamin D and calcitriol can`t be effective on reducing the inflammation. Due to the great possibility of genetic involvement in Vitamin D's effect on respiratory disease and the high prevalence of Vitamin D deficiency in Iran, it is suggested to conduct clinical trials among the Iranian people and evaluate the efficacy of Vitamin D [33].

Our study had some limitations. First most of our patients were Vitamin D sufficient so we couldn’t determine the effect of short term treatment in patient Vitamin D deficiency (which may be more effective). Longer duration with larger sample size study in vitamin D deficient patients with COPD suggests. Second our study was too short to follow the patient for readmission so we recommend study the effect of calcitriol on the frequency of hospitalization. Third as this study were done in normocalcemic patient we had chosen the lowest dosage of calcitriol, higher doses of calcitriol can be used in future studies.

Given the inefficacy of current treatments in COPD, there is still a need for further randomized control trials with vitamin D supplementation. In such randomized controlled trials in which vitamin D treatment is only based on bone maintenance, defining the optimal serum levels for 25-OHD in this disease as reference for other calcemic endpoints is a challenging task. Future clinical trials are suggested to evaluate the impact of higher levels of vitamin D and especially calcitriol on lung function during exacerbation. We suggest investigating this correlation in vitamin D deficient patients.

## Conclusion

Short term treatment with either calcitriol or 25(OH) _2_Vit D didn`t changed FEV1 or FVC in vitamin D sufficient patients with COPD exacerbation; nevertheless it can provide clinical benefit.

## Abbreviations

Alb: Albumin
Ca: Calcium
25OH-D: Vitamin D
1,25OH-D: 1,25Vitamin D
CRP: C reactive protein
Calcitriol: Calcitriol
